# Synthesis and Antimicrobial Activity of Long-Chain 3,4-Epoxy-2-alkanones

**DOI:** 10.3797/scipharm.1009-02

**Published:** 2010-10-09

**Authors:** William F. Wood

**Affiliations:** Department of Chemistry, Humboldt State University, 1 Harpst St., Arcata, California, 95521, U.S.A

**Keywords:** Antifungal activity, 3,4-Epoxy-2-alkanones, *Propionibacterium*, *Trichophyton*

## Abstract

3,4-Epoxy-2-dodecanone, a major component in the preorbital gland of the African grey duiker (*Sylvicapra grimmia*), showed antimicrobial activity in preliminary tests. The C_11_ to C_17_ homologues of this compound were prepared and their activity against several pathogenic dermal bacteria and fungi was tested. 3,4-Epoxy-2-dodecanone and 3,4-epoxy-2-tridecanone inhibited the growth of *Trichophyton mentagrophytes* at 25 μg/mL. Moderate inhibition of the growth of the bacteria *Propionibacterium acnes* and the lipophilic yeast, *Pityrosporum ovale*, was seen for several of the homologues.

## Introduction

Compounds with antimicrobial activity have been documented from the skin and skin glands of a number of mammalian species. (*E*)-3-Tridecen-2-one (**1c**, n=7) the major compound from interdigital glands of the black-tailed deer (*Odocoileus hemionus columbianus*) has significant antimicrobial activity [1–3]. Nonanal, octanal and heptanal were found in the hair of Mexican free-tailed bats (*Tadarida brasiliensis mexicana*) at concentrations known to inhibit the growth of pathogenic skin fungi [4]. Hair from the reticulated giraffe (*Giraffa camelopardalis reticulata*) also has nonanal, octanal and heptanal at high concentrations and *p*-cresol, indole, tetradecanoic acid and hexadecanoic acid, compounds that inhibit the growth of pathogenic skin fungi and bacteria [5]. Several antimicrobial long-chain alcohols were found in the interdigital glands of the American pronghorn (*Antilocapra americana*) [6]. In search of new antimicrobials from animal skin glands, 3,4-epoxy-2-dodecanone (**2b**, n=6), a major component in the African grey duiker’s (*Sylvicapra grimmia*) preorbital skin gland [7] was prepared and assayed. It showed antifungal activity in preliminary tests, so the C_11_ to C_17_, 3,4-epoxy-2-alkanones (**2a–2g**, Scheme 1) were prepared and tested for antimicrobial activity. The activity of these compounds was compared to antimicrobial activity of the (*E*)-3-alken-2-ones (**1a–1g**) from which they were prepared [3].

## Results and Discussion

### Chemistry

Naturally occurring, long-chain 3,4-epoxy-2-alkanones have infrequently been reported in the chemical literature. α,β-Epoxyketones are easily prepared by reaction of hydrogen peroxide and sodium hydroxide with α,β-unsaturated ketones [7, 8]. This method was used with previously prepared long-chain (*E*)-3-alken-2-ones [3] to make the 3,4-epoxy-2-alkanones in this report.

### Antimicrobial Activity

The minimum inhibitory concentrations (MIC) for *Trichophyton mentagrophyte*s, *Propionibacterium acnes* and *Pityrosporum ovale* with the 3,4-epoxy-2-alkanones are listed in Table 1. 3,4-Epoxy-2-dodecanone (**2b**), the compound from the grey duiker, and 3,4-epoxy-2-tridecanone (**2c**) inhibited the growth of *T. mentagrophyte*s at the same concentration, 25 μg/mL. Activity against this fungus decreased at longer or shorter chain lengths. For the bacterium, *P. acnes*, the C_11_ to C_15_ homologues (**2a–2e**) were active at 100 μg/mL, but the C_16_ and C_17_ homologues (**2f** and **2g**) were inactive. With the lipophilic yeast, *P. ovale*, the shorter chain-length compounds [C_11_ to C_16_ (**2a–2f**)] were not active at 800 μg/mL, but the C_17_ compound (**2g**) was active at 200 μg/mL. No activity was observed with the bacterium *Staphylococcus aureus* and the yeast *Candida albican*s. For all microorganisms tested, a control using the dissolution medium, dimethylformamide (DMF), had no activity.

Table 2 has the comparable inhibitory activity of the (*E*)-3-alken-2-ones (**1a–1g**) [3], the compounds that were used to prepare the 3,4-epoxy-2-alkanones in this report. The inhibition of *T. mentagrophyte*s for both sets of compounds is similar, with activity for the C_11_ to C_14_ homologues, which decreases starting with the C_15_ compound. This suggests that these compounds have a similar mode of action with *T. mentagrophyte*s. The inhibition of growth for *P. acnes* is quite different for these two series of compounds. The (*E*)-3-alken-2-ones (**1a–1g**) have activity increase with increasing carbon chain length. The shorter chain-length 3,4-epoxy-2-alkanones are not as active as the corresponding (*E*)-3-alken-2-ones and the longer homologues do not inhibit *P. acnes*. This suggests that these compounds have a different mode of action. The 3,4-epoxy-2-alkanones were almost inactive with the yeast *P. ovale*. Only the C_17_ compound showed slight activity. The activity of the (*E*)-3-alken-2-ones against *P. ovale* is moderate for the C_11_ to C_14_ homologues and ceases at longer chain lengths (C_16_ to C_17_). This also suggests that these compounds have different modes of action. Both types of compound were not active against *S. aureus*.

The epoxide and double bond at the 3,4-position of the 3,4-epoxy-2-tridecanone (**2c**) and (*E*)-3-tridecen-2-one (**1c**) are important to the activity of these two compounds, since 2-tridecanone was inactive on bioassay with these microorganisms [2]. Thus, a possible mode of action for these two compounds may be an irreversible inhibition of an enzyme due to a nucleophilic attack at the 4-position. With (*E*)-3-tridecen-2-one this would be a 1,4-addition (Michael addition) and with 3,4-epoxy-2-tridecanone an epoxide opening. Other homologous 2-alkanones have not been tested, so a comparison with other compounds in this report was not possible.

## Experimental

### Chemistry

^1^H-NMR and ^13^C-NMR spectra were recorded in CDCl_3_ on a JEOL ECX-300. Mass spectral data were collected on a Hewlett-Packard GCD Plus. For antimicrobial assays and spectral analysis, compounds were purified by preparative chromatography with a Gow-Mac Series 580 using a 2 m by 5 mm column that had a 3% OV101 liquid phase. Sample purity for antimicrobial assays was confirmed by GC-MS analysis. The GC-MS analyses were performed on a CH_2_Cl_2_ solution of the purified sample in a splitless mode (0.5 min), using a Hewlett-Packard GCD Plus fitted with a 30-m × 0.25-mm cross-linked phenyl methyl silicone capillary column (HP-5MS). The gas chromatograph was programmed so the oven temperature was kept at 40°C for 4 min, then increased to a final temperature of 325°C at a rate of 30°C/min and held at this temperature for 5 min.

### Synthesis of 3,4-Epoxy-2-alkanones (2a–2g)

To a stirred solution (20°C) of 0.005 mole of the appropriate (*E*)-3-alken-2-one [3] in 20 mL of methanol and 1.5 mL of 30% H_2_O_2_, was added 0.4 mL of 6 *M* NaOH, dropwise over 1 minute. After stirring for 10 min at RT, the reaction was quenched with 25 mL of water and then extracted into 2 × 25 mL of diethyl ether. The ether solution was washed with 2 × 50 mL water, dried over sodium sulfate and concentrated *in vacuo*. The compounds were purified by preparative gas chromatography.

#### 3,4-Epoxy-2-undecanone (1-(3-heptyloxiran-2-yl)ethanone, **2a**)

Yield 64%. Liquid, 300 MHz ^1^H-NMR (CDCl_3_) δ = 3.14 (d, 1H, J=4.8Hz), 3.03 (dt, 1H, J=12.9 Hz, J=4.8 Hz), 2.02 (s, 3H), 1.58 (m, 2H), 1.42 (m, 2H),1.23 (m, 8H) and 0.84 (t, 3H); 75 MHz ^13^C-NMR (CDCl_3_) δ = 206.18, 59.89, 58.09, 31.71, 31.64, 29.15, 29.03, 25.71, 24.31, 22.54, and 14.00; and EI-MS m/z = 100(6), 85(98), 82(7), 81(15), 69(8), 67(9), 57(30), 55(23), 43(100) and 41(27).

#### 3,4-Epoxy-2-dodecanone (1-(3-octyloxiran-2-yl)ethanone, **2b**)

Yield 70%. Liquid, 300 MHz ^1^H-NMR (CDCl_3_) δ = 3.14 (d, 1H, J=4.8Hz), 3.03 (dt, 1H, J=12.9 Hz, J=4.8 Hz), 2.02 (s, 3H), 1.58 (m, 2H), 1.42 (m, 2H),1.23 (m, 10H) and 0.84 (t, 3H); 75 MHz ^13^C-NMR (CDCl_3_) δ = 206.12, 59.89, 58.07, 31.76, 31.72, 29.35, 29.21, 29.09, 25.72, 24.29, 22.58, and 14.03; and EI-MS m/z = 100(7), 85(100), 82(7), 81(15), 69(14), 67(9), 57(31), 55(20), 43(91) and 41(26).

#### 3,4-Epoxy-2-tridecanone (1-(3-nonyloxiran-2-yl)ethanone, **2c**)

Yield 80%. Liquid, 300 MHz ^1^H-NMR (CDCl_3_) δ = 3.14 (d, 1H, J=4.8 Hz), 3.03 (dt, 1H, J=12.9 Hz, J=4.8 Hz), 2.02 (s, 3H), 1.58 (m, 2H), 1.42 (m, 2H),1.23 (m, 12H) and 0.84 (t, 3H); 75 MHz ^13^C-NMR (CDCl_3_) δ = 206.12, 59.90, 58.07, 31.80, 31.73, 29.41, 29.37, 29.25, 29.20, 25.71, 24.30, 22.61, and 14.04; and EI-MS m/z = 95(19), 85(100), 81(7), 81(12), 69(13), 67(10), 57(29), 55(20), 43(90) and 41(26).

#### 3,4-Epoxy-2-tetradecanone (1-(3-decyloxiran-2-yl)ethanone, **2d**)

Yield 79%. Liquid, 300 MHz ^1^H-NMR (CDCl_3_) δ = 3.14 (d, 1H, J=4.8 Hz), 3.03 (dt, 1H, J=12.9 Hz, J=4.8 Hz), 2.02 (s, 3H), 1.58 (m, 2H), 1.42 (m, 2H),1.23 (m, 14H) and 0.84 (t, 3H); 75 MHz ^13^C-NMR (CDCl_3_) δ = 206.12, 59.88, 58.07, 31.82, 31.72, 29.50, 29.43, 29.38, 29.24, 29.20, 25.70, 24.28, 22.62, and 14.03; and EI-MS m/z = 95(14), 85(87), 83(10), 81(11), 69(11), 67(10), 57(28), 55(22), 43(100) and 41(31).

#### 3,4-Epoxy-2-pentadecanone (1-(3-undecyloxiran-2-yl)ethanone, **2e**)

Yield 80%. Liquid, 300 MHz ^1^H-NMR (CDCl_3_) δ = 3.14 (d, 1H, J=4.8 Hz), 3.03 (dt, 1H, J=12.9 Hz, J=4.8 Hz), 2.02 (s, 3H), 1.58 (m, 2H), 1.42 (m, 2H),1.23 (m, 16H) and 0.84 (t, 3H); 75 MHz ^13^C-NMR (CDCl_3_) δ = 206.19, 59.90, 58.09, 31.85, 31.73, 29.60, 29.56, 29.39, 29.30, 29.28, 29.21, 25.72, 24.30, 22.63, and 14.06; and EI-MS m/z = 95(14), 85(87), 83(9), 81(13), 69(12), 67(11), 57(29), 55(23), 43(100) and 41(31).

#### 3,4-Epoxy-2-hexadecanone (1-(3-dodecyloxiran-2-yl)ethanone, **2f**)

Yield 74%. Liquid, 300 MHz ^1^H-NMR (CDCl_3_) δ = 3.14 (d, 1H, J=4.8 Hz), 3.03 (dt, 1H, J=12.9 Hz, J=4.8 Hz), 2.02 (s, 3H), 1.58 (m, 2H), 1.42 (m, 2H),1.23 (m, 18H) and 0.84 (t, 3H); 75 MHz ^13^C-NMR (CDCl_3_) δ = 206.18, 59.91, 58.08, 31.85, 31.75, 29.60, 29.57, 29.45, 29.40, 29.29, 29.21, 25.74, 24.32, 24.21, 22.66, and 14.06 and EI-MS m/z = 95(13), 85(77), 83(10), 81(12), 69(12), 67(10), 57(28), 55(23), 43(100) and 41(31).

#### 3,4-Epoxy-2-heptadecanone (1-(3-tridecyloxiran-2-yl)ethanone, **2g**)

Yield 74%. Solid, 300 MHz ^1^H-NMR (CDCl_3_) δ = 3.14 (d, 1H, J=4.8 Hz), 3.03 (dt, 1H, J=12.9 Hz, J=4.8 Hz), 2.02 (s, 3H), 1.58 (m, 2H), 1.42 (m, 2H),1.23 (m, 20H) and 0.84 (t, 3H); 75 MHz ^13^C-NMR (CDCl_3_) δ = 206.19, 59.92, 58.08, 31.89, 31.75, 29.57, 29.57, 29.57, 29.45, 29.40, 29.29, 29.21, 25.74, 24.32, 24.22, 22.66, and 14.09; and EI-MS m/z = 95(12), 85(66), 83(10), 81(11), 69(13), 67(10), 57(29), 55(22), 43(100) and 41(32).

### Antimicrobial Assay

The 3,4-epoxy-2-alkanones were screened against some mammalian skin pathogens: the bacteria *Staphylococcus aureus*, ATCC 12598, *Propionibacterium acnes* ATCC 11827; and the fungi *Candida albican*s ATCC 24433, *Pityrosporum ovale* ATCC 14521 and *Trichophyton mentagrophytes* ATCC 18748. The minimum inhibitory concentration (MIC in μg/mL) of these 3,4-epoxy-2-alkanones was done using a two-fold serial broth dilution. Two replicates were done with each microorganism and the MIC was determined as the lowest concentration for each compound at which no growth was observed. The highest concentration used in these tests was 800 μg/mL.

Each test compound (80 mg) was dissolved in 1.0 mL of dimethylformamide (DMF) and 30 μL of this stock solution was dissolved in 3 mL of the applicable medium. Two-fold serial dilution of the resulting 800 µg/mL solution, gave solutions of 400, 200, 100, 50, 25, 12.5, 6.25, and 3.12 μg/mL. In addition, for each microorganism, a control of 30 μL DMF in 3 mL of medium was tested. Finally, to each of the diluted test solutions was added a 30 μL sample of microorganism culture. The cultures of *S. aureus*, *P acnes* and *C. albican*s were examined for turbidity (OD at 660 nm). *P. ovale* was examined visually for growth at two days and *T. mentagrophytes* at five days.

*T. mentagrophytes* lyophile was reconstituted in sterile water and grown on media containing 1 % peptone and 4 % glucose. A seven-day-old plate of well sporulating pure culture was washed and resuspended in 1 L of media. A 30 μL sample of this culture was dispensed into each 3 mL sample of the diluted test solutions (1 % peptone and 4 % glucose). The 13 × 100 mm glass culture tubes with Morton cap closures were incubated at 30°C.

*P. acnes* lyophile was reconstituted in sterile media and grown in screw cap tubes at 37°C in media containing 0.8% nutrient broth, 0.5% yeast extract, 0.1% glucose to which 1% Oxyrase (Oxyrase, Inc.) was added after autoclaving. At two days, a 30 μL sample of this culture was mixed with each diluted test solution [0.8% nutrient broth (BBL), 0.5% yeast Extract (Difco) and 0.1% glucose (NYG broth)]. The 13 × 100 mm glass culture tubes with Morton cap closures were incubated at 37°C.

*P. ovale* was shake-cultured in 1% bactopeptone (Difco), 0.5% yeast extract, 1% glucose and 0.1% corn oil for two days at 30°C. *C. albican*s was shake-cultured in a 2.5 % malt extract broth (BBL) for two days at 30°C. *S. aureus* was incubated for two days at 37°C in a culture media containing 0.8% nutrient broth (BBL), 0.5% yeast Extract (Difco) and 0.1% glucose (NYG broth).

## Conclusions

Preorbital glands are common among ungulates, which usually mark twigs and grass with their contents as a chemical cue to other members of the species [9, 10]. Because of scent marking in cases like this, almost all animal skin glands are usually referred to as “scent glands.” While the semiochemical function of these animal skin glands has been the focus of research in this area, a further function of these glands may be to produce antimicrobial compounds against dermal pathogens. Antimicrobial compounds found in these glands may be biosynthesized by the animal itself, or by microbes that live in these glands. Prospecting for antimicrobial compounds from animal skin glands followed by synthesis of appropriate analogues is an area of drug discovery that has been largely unexplored and has the potential of producing new antimicrobial agents against pathogenic dermal organisms.

## Figures and Tables

**Sch. 1.**
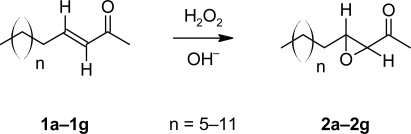
Preparation of 3,4-epoxy-2-alkanones

**Tab. 1.** Minimum inhibitory concentration (MIC) of 3,4-epoxy-2-alkenones (μg/mL)

**Compound**	***T. mentagrophytes***	***P. acnes***	***P. ovale***
3,4-Epoxy-2-undecanone (**2a**)	50	100	>800
3,4-Epoxy-2-dodecanone (**2b**)	25	100	>800
3,4-Epoxy-2-tridecanone (**2c**)	25	100	>800
3,4-Epoxy-2-tetradecanone (**2d**)	50	100	>800
3,4-Epoxy-2-pentadecanone (**2e**)	400	100	>800
3,4-Epoxy-2-hexadecanone (**2f**)	>800	>800	>800
3,4-Epoxy-2-heptadecanone (**2g**)	>800	>800	200

**Tab. 2.** Minimum Inhibitory Concentration (MIC) of 2-Alken-3-ones (μg/mL)

**Compound**	***T. mentagrophytes***	***P. acnes***	***P. ovale***
2-Undecen-3-one (**1a**)	100	50	100
2-Dodecen-3-one (**1b**)	100	25	100
2-Tridecen-3-one (**1c**)	25	12.5	100
2-Tetradecen-3-one (**1d**)	12.5	12.5	100
2-Pentadecen-3-one (**1e**)	800	6.25	400
2-Hexadecen-3-one (**1f**)	>800	3.13	>800
2-Heptadecen-3-one (**1g**)	>800	3.13	>800
